# Structure–Property Relationship and Multiple Processing Studies of Novel Bio-Based Thermoplastic Polyurethane Elastomers

**DOI:** 10.3390/ma16186246

**Published:** 2023-09-17

**Authors:** Joanna Smorawska, Marcin Włoch, Ewa Głowińska

**Affiliations:** Department of Polymers Technology, Faculty of Chemistry, Gdansk University of Technology, 11/12 Gabriel Narutowicza Street, 80-233 Gdansk, Poland; s177280@student.pg.edu.pl (J.S.); marcin.wloch@pg.edu.pl (M.W.)

**Keywords:** bio-based polyurethanes, mechanical recycling, sustainable materials, reprocessing, circular economy

## Abstract

Currently, the growing demand for polymeric materials has led to an increased need to develop effective recycling methods. This study focuses on the multiple processing of bio-based thermoplastic polyurethane elastomers (bio-TPUs) as a sustainable approach for polymeric waste management through mechanical recycling. The main objective is to investigate the influence of two reprocessing cycles on selected properties of bio-TPUs. Two series of bio-based TPUs were synthesized via a solvent-free two-step method with the use of hexamethylene diisocyanate or hexamethylene diisocyanate/partially bio-based diisocyanate mixtures, bio-based poly(triamethylene ether) glycol, and bio-based 1,3 propanediol. Both the raw bio-TPUs and those subjected to two reprocessing cycles were examined with respect to their chemical, physical, thermal, thermomechanical, and mechanical properties. The conducted research revealed that reprocessing led to changes in the phase separation between the hard and soft segments, thereby affecting the bio-TPUs’ properties. Both series of materials showed similar chemical structures regardless of reprocessing (slight changes were observed in the range of carbonyl peak). The thermal properties of TPUs exhibited slight differences after each reprocessing cycle, but generally, the non-processed and reprocessed bio-TPUs were thermally stable up to about 300 °C. However, significant differences were observed in their mechanical properties. The tensile strength increased to 34% for the twice-reprocessed bio-TPUs, while the elongation at break increased by ca. 200%. On the other hand, the processing cycles resulted in a decrease in the hardness of both bio-TPU series (ca. 3–4 °ShA). As a result, the prepared bio-TPUs exhibited characteristics that were closer to those of the sustainable materials model, promoting the circular economy of plastics, with environmental benefits arising from their recyclability and their high content of bio-based monomers (78.4–78.8 wt.%).

## 1. Introduction

In this day and age, a noticeable interest in thermoplastic polyurethane elastomers (TPUs) can be observed. The market size for TPUs was estimated to reach USD 3.2 billion in 2022, and is projected to reach USD 4.6 billion in 2027, representing a compound annual growth rate (CAGR) of 7.1% [1]. The increasing demand for TPU materials is a result of their numerous advantages. For instance, TPUs have been applied both in everyday life and in the industry, and are used in the automotive sector, furniture, footwear, medical devices, polymer films, and thermal insulation [2,3]. With the increase in TPU production, there has also been a corresponding increase in post-processing and postconsumer waste. In light of the growing emphasis on environmental concerns and proper waste management, TPUs show promise as potentially eco-friendly plastics. Their ability to be used in hot-melt processing methods makes mechanical recycling and reprocessing feasible, contributing to their suitability in circular economy processes.

**Figure 1:**
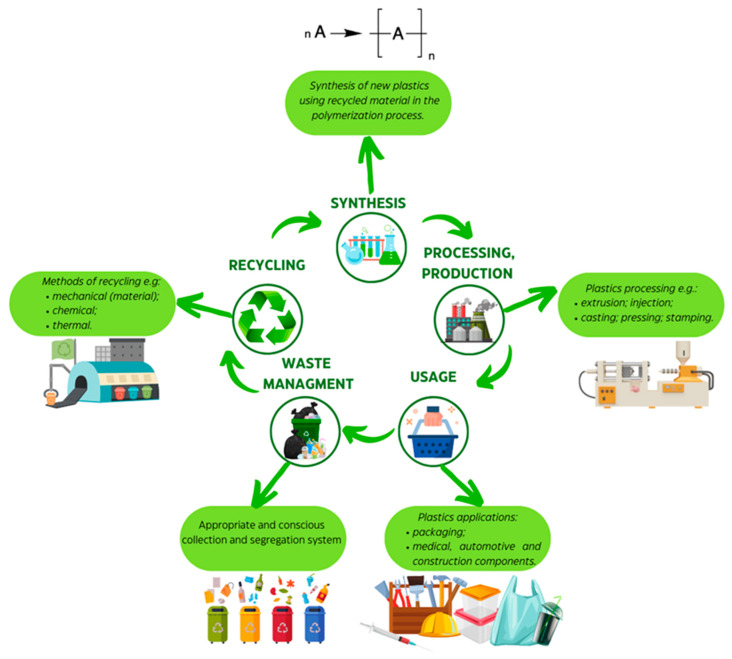
Scheme showing the circular plastics life with selected examples of processing or operation at each particular step. The life of circular plastics represents a sustainable model in which plastics remain in circulation for a longer period through their reuse and recycling [11].

Polyurethane waste management typically involves feedstock or mechanical recycling methods. The current focus in recycling is on achieving a circular plastics life (Figure 1). The recycling of polyurethane stream waste depends on the type; for example, thermosets or thermoplastics can be recycled via mechanical and/or chemical methods [4]. The resulting products can be reused in PU synthesis (liquid products), incorporated into polymer blends (solid products), or used as polymer fillers. Chemical recycling, involving processes like glycolysis, acidolysis, hydrolysis, or glycerolysis, has become one of the most developed recycling methods for polyurethanes [5,6,7,8,9]. The end products of chemical recycling depend on the specific chemicals used and the polyurethane composition [10].

Mechanical recycling is a commonly known method for polymeric waste management and is based on physical processes and treatments [12]. The main idea of the mechanical recycling of polymers is the reuse of post-processing or postconsumer waste without significantly changing their chemical structures. The literature highlights several advantages of mechanical recycling, including simplified processing (sorting, cleaning, grinding, and reusing) due to the highly economical and ecologically efficient approach in which raw materials are substituted by recycled TPUs [12,13].

However, successful mechanical recycling is limited to a few cycles using minor fractions of the purest and cleanest waste stream. It is for this reason that mechanical recycling can be limited in terms of the purity of the waste stream if the recycling product is intended for high-end applications. Postconsumer plastics exhibit a lower amount of properties, and usually contain a lot of additives such as fillers, plasticizers, or pigments [14], making reprocessing more challenging. The variable chemical structure of polyurethane segments demands for recycling procedures to be individually designed.

Unlike typical thermoplastic materials like polyolefins, the mechanical recycling of polyurethanes is a more complex process. Polyurethanes can differ in chemical structure due to the wide range of monomers used for their synthesis. Therefore, the mechanical recycling of post-processing waste might be of particular interest. In a literature review, examples of mechanical recycling conducted on polyurethane foams were found. After the mechanical regrinding of PU waste, recycled and reground wastes can be used as fillers in new formulations, usually with the use of binders [4,12,15,16].

Calvo-Correas et al. [13] conducted a study on ear tags in the livestock sector made of TPU via mechanical recycling. Multiple extrusion and injection molding cycles were performed, revealing that multiple reprocessing cycles might lead to TPU degradation. Based on the chemical structure and thermal and mechanical analyses, it was revealed that the use of more than a single reprocessing cycle might lead to the degradation of TPU waste. These findings were confirmed, for example, via GPC analysis, which revealed a decrease in the molecular weight of the TPU or a decrease in the tensile properties after each processing cycle. Nevertheless, the authors proved that the recycled TPU is suitable for 3D printing and electrospinning techniques, which is a huge advantage of this material.

Wölfel et al. [17] studied the recycling and reprocessing of thermoplastic polyurethanes based on polytetramethylene glycol (PTMG), methylendiphenylisocyanate (MDI), and 1,4-butanediol (BDO). The hard segments content was around 30 wt.%. Recycling and several reprocessing cycles were carried out via extrusion. The recycled material derived from each cycle was used for nonwoven production with the use of a melt blowing line. The authors noticed that after each reprocessing cycle, the TPUs become more brittle, and their molecular weights decreased, as well as their tensile properties. Based on the obtained results, Wölfel et al. suggested the existence of two separate degradation regimes for the soft and hard segments of TPUs.

Plummer et al. [18] investigated the recyclability of a new thermoplastic polyurethane powder in the laser sintering process. The elastomer bed powder was recycled four times without significant changes in the thermal properties, particle size, or tensile properties. The authors noticed a slight decrease in the tensile strength and elongation at break with an increasing number of reprocessing cycles, which can be explained by a reduced chain length of the macromolecules. 

In another study, thermoplastic polyurethane waste derived from orthopedic devices was used to prepare nanocomposites. For this purpose, low-temperature biodegradable polycaprolactone-based thermoplastic polyurethane (rTPU) was filled with modified montmorillonite nanoclays. In this case, reprocessing through two-roll milling led to an increase in the melt flow index and a decrease in the mechanical properties and molecular weight of the rTPU. These effects were caused by chain scission and cross-linking. In general, the mentioned properties depended on the reprocessing time [19].

In our previous work [20], we discussed the chemical structure and the thermal and mechanical properties of petrochemical-based thermoplastic poly(ester-urethanes) subjected to double processing (after their synthesis). The TPUs were obtained using bio-based glycols and were characterized based on different hard segment contents. The study revealed a decrease in the formation of hydrogen bonds and a subsequent reduction in the tensile strength and hardness upon subjecting the materials to a second processing cycle. 

The present study aims to demonstrate the feasibility of mechanically recycling novel bio-based TPUs through multiple processing cycles. Raw (non-reprocessed), once-reprocessed, and twice-reprocessed TPUs are studied in this work in terms of their chemical structure and their thermal, thermomechanical, mechanical, and processing properties. The novelty of this work is the study of the influence of partially bio-based isocyanate on the mentioned properties. 

## 2. Materials and Methods

### 2.1. Synthesis Procedure of Bio-Based Thermoplastic Polyurethanes 

**Figure 2:**
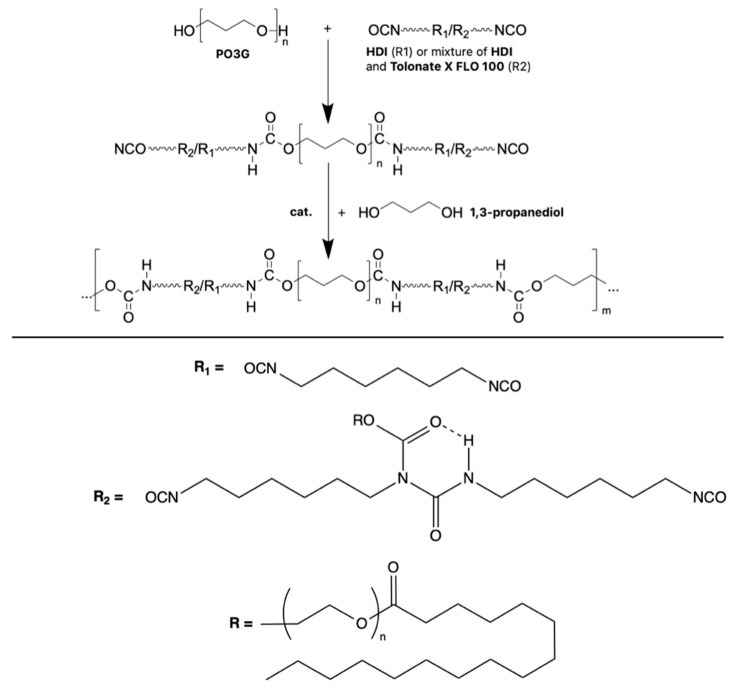
Illustrative scheme of bio-TPUs’ synthesis via prepolymer method.

The synthesis of thermoplastic polyurethane elastomers was carried out via a two-step method, known as the prepolymer method. In the first step, each prepolymer was synthesized using polyether polyol (poly(trimethylene) glycol), PO3G, M_n_~2000 with the trade name of VELVETOL^®^H2000 (Allesa, Frankfurt, Germany), and diisocyanate (hexamethylene diisocyanate, HDI; NCO content, ~49.9%; Vencorex, Saint-Priest, France) or a diisocyanate mixture containing HDI and partially bio-based diisocyanate (Tolonate™ X FLO 100; NCO content, ~12.3%; Vencorex, Saint-Priest, France). The 90 wt.% of NCO groups in the diisocyanate mixture was provided via HDI component, while 10 wt.% was introduced by Tolonate™ X FLO 100. The prepolymerization reaction was conducted for 3 h at a temperature of 85 °C under vacuum. The free NCO content in each prepolymer was determined using the titration method following ASTM D 2572-97 [21]. The free NCO contents were 8.20 +/− 0.1 wt.% and 8.22 +/− 0.1 wt.% for prepolymers series 100H and 90H, respectively. Next, each prepolymer was extended using a low-molecular-weight chain extender, specifically bio-based 1.3-propanediol (DuPont, Wilmington, DE, USA). This reaction was catalyzed using dibutyltin dilaurate (DBTDL, Sigma-Aldrich, Poland division). All TPU samples were prepared with a final molar ratio of isocyanate group to hydroxyl group (NCO/OH) equal to 0.95. Molded bio-TPUs were annealed at 100 °C for 24 h in a laboratory oven to achieve fully cured materials. Illustrative scheme of bio-TPUs’ synthesis is shown in Figure 2. 

### 2.2. Processing of Bio-Based Thermoplastic Polyurethanes

**Figure 3:**
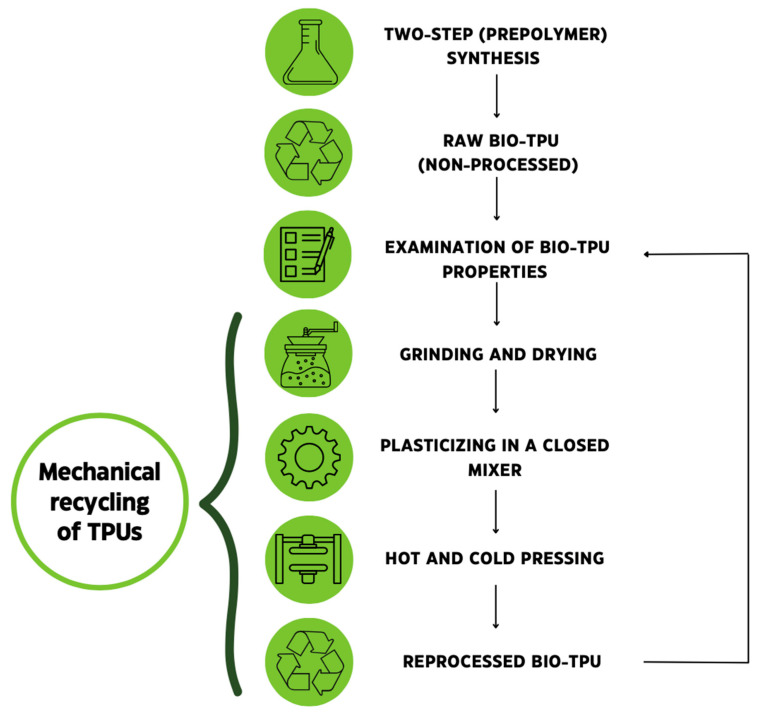
Illustrative scheme of experimental part including bio-TPU synthesis, properties investigation, and mechanical reprocessing of bio-TPUs.

Processing of bio-based thermoplastic polyurethanes TPUs was conducted using a closed mixer (using the melt mixing method) and hydraulic press was used for sample molding. Firstly, the grounded TPUs were plasticized and homogenized for 10 min in the closed mixer at approximately 145–150 °C (depending on the bio-TPUs’ compositions). In the second step, the processed bio-TPU samples were hot-pressed for 5 min in a hydraulic press at a pressure of 5 MPa. After hot pressing, the samples were cold-pressed until they reached room temperature. For two series of bio-TPU, the processing cycle was repeated twice. It is worth mentioning that after each processing cycle, the materials were examined in order to determine their chemical structure and selected properties. Figure 3 presents the illustrative scheme of the idea of reprocessing synthesized bio-TPUs. Codes of the samples and content of each isocyanate in the prepolymerization reaction, contents of hard segments and bio-based components, and sample pictures are presented in Table 1.

### 2.3. Measurements

#### 2.3.1. Fourier-Transform Infrared Spectroscopy (FTIR)

Fourier-Transform Infrared Spectroscopy was performed using a Nicolet FTIR 8700 spectrophotometer (Thermo Electron Corporation, Waltham, MA, USA) equipped with an attenuated total reflectance accessory. The resolution of the spectrophotometer was equal to 4 cm^−1^. Spectra were registered at room temperature over a wavenumber ranging from 500 to 4500 cm^−1^. Each spectrum was acquired with 64 scans. 

#### 2.3.2. Thermogravimetric Analysis (TGA)

Thermogravimetric analysis (TGA) was carried out using a NETZSCH TG 209F3 analyzer (NETZSCH, Selb, Germany). The measurements were performed under a nitrogen atmosphere at a heating rate of 10 K/min with temperature ranging from 35 to 800 °C. The weight of each sample was ca. 10 mg.

#### 2.3.3. Differential Scanning Calorimetry (DSC)

Differential scanning calorimetry was carried out by using a DSC 204 F1 Phoenix^®^ calorimeter (NETZSCH, Selb, Germany). Each sample with a weight of ca. 10 mg was placed in a closed aluminum pan and heated twice from −80 °C to 240 °C at a scanning rate of 10 °C/min and cooled once from 240 °C to −80 °C at a scanning rate of 5 °C/min. Measurements were taken using nitrogen as a purge gas (20 mL/min).

#### 2.3.4. Dynamic Mechanical Analysis (DMTA)

Dynamic mechanical analysis of bio-TPU was performed using a DMA Q800 Analyzer (TA Instruments, New Castle, DE, USA). Measurements were carried out according to ASTM D4065:2012 [22]. Measurements were taken in the three-point bending mode at an operating frequency of 10 Hz. The measurements were made at temperatures ranging from −100 to 150 °C with a heating rate of 3 °C/min.

#### 2.3.5. Melt Flow Index (MFI)

The melt flow index test was carried out using a Zwick/Roell plastometer (Zwick Roell Group, Ulm, Germany) according to EN ISO 1133:2006 [23]. Tests were taken at temperatures ranging from 135 to 150 °C and loads of 2.16 and 5 kg, and depended on bio-TPU composition and number of processing cycles.

#### 2.3.6. Tensile Properties

Static tensile test was carried out using a Zwick/Roell Z020 universal testing machine (Zwick Roell Group, Ulm, Germany) according to EN ISO 527-1:2012 and 527-2:2012 [24,25]. Dumbbell-shaped samples (type 5A) were tested with a crosshead speed of 100 mm/min. Measurements were taken at room temperature. Properties (tensile strength, elongation at break, and permanent elongation after break) during the test were average values from five independent tests.

#### 2.3.7. Hardness

Hardness of bio-TPUs was estimated following PN-EN ISO 868:2005 [26]. Shore A durometer-Zwick/Roell HPE (Zwick Roell Group, Ulm, Germany) was used. Measurements were taken at room temperature. Ten measurements were taken per sample.

#### 2.3.8. Density

Density of bio-TPUs was determined according to PN-EN ISO 1183-1:2019-05 [27]. Measurements were taken using electronic analytical balance AS 310/X (RADWAG, Radom, Poland) equipped with a software for density calculation. The measurement was carried out using the hydrostatic method; the weight of the sample was measured in the air and after immersion in methanol. Temperature of immersion liquid was 23 °C.

## 3. Results and Discussion

### 3.1. Chemical Characterization of Non-Processed (Raw) and Reprocessed Bio-TPUs

#### Fourier-Transform Infrared Spectroscopy (FTIR)

In this work, FTIR spectroscopy was used to identify the characteristic chemical groups present in the chemical structure of the prepared thermoplastic bio-polyurethanes. Identification was performed on the basis of the position of the absorption bands in the FTIR spectrum. On the other hand, spectroscopy was performed to follow the structural changes that might occur during the reprocessing of the material.

**Figure 4:**
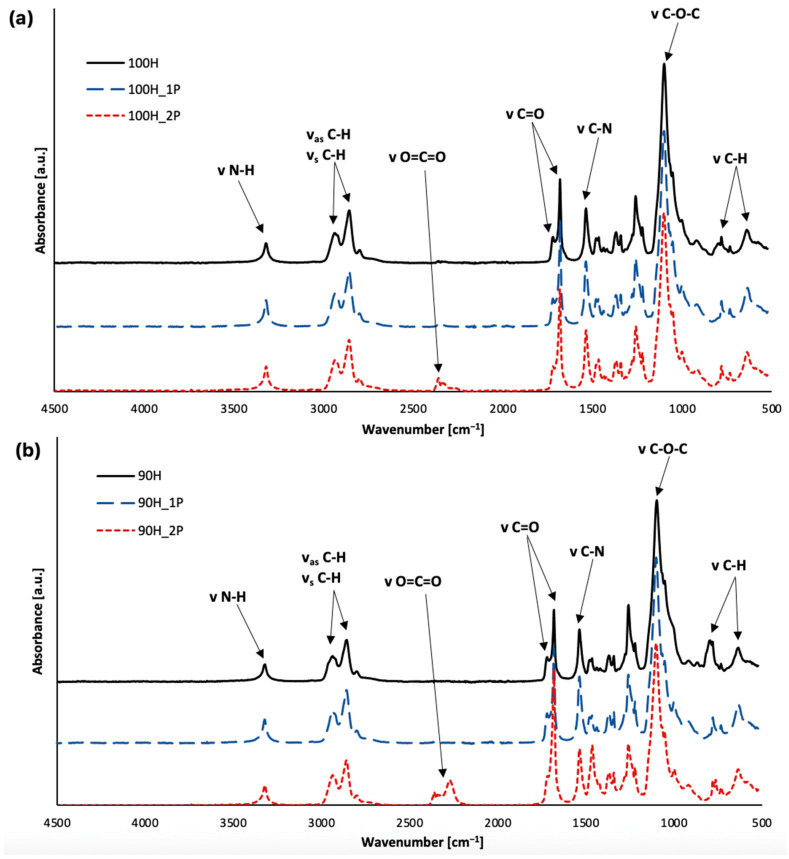
FTIR spectra of bio-TPUs: (**a**) 100H and (**b**) 90H before and after two cycles of processing.

Figure 4a,b shows the FTIR spectra for the non-processed 100H and 90H material series and after the first and second cycles of reprocessing. Comparing each spectrum, there are no significant changes in their course. For all of the bio-TPUs, in the wavenumber range from 2250 to 2270 cm^−1^, a signal corresponding to the isocyanate NCO group is not visible, which indicates that the reactions of all the unbound NCO groups with the OH groups were completed. On the other hand, if we consider the spectra of the reprocessed materials, the absence of free NCO groups suggests that the processing did not cause the depolymerization of the bio-TPU with the release of the isocyanate component. The formation of the urethane group (NHC(O)O) is proven by a band in the wavenumber range of 3313–3320 cm^−1^, which is attributed to the vibrations of the N-H group. The next characteristic for the urethane group band was found as a peak related to the vibration of the C-N group at 1540 cm^−1^ and multiple peaks related to the stretching vibration of C=O ranging from 1683 to 1712 cm^−1^, which indicated hydrogen-bonded C=O and free carbonyl. The valence vibration of C-O-C was observed in the range of 1106–1097 cm^−1^ and resulted from the presence of ether groups in the bio-polyol. Signals representing the asymmetric (2930–2915 cm^−1^) and symmetric (2850–2860 cm^−1^) vibrations of the methylene group can be found in all spectra [28,29,30,31]. The differences in the wavenumber ranges of each characteristic bond vibrations for the bio-TPU are included in Table 2.

As was mentioned previously, the analysis of the chemical structure of the bio-TPUs showed slight differences between the samples before and after processing. Changes in the range of the carbonyl bond (C=O) vibration band for the materials after each processing cycle are visible. In each spectrum of both series of reprocessed materials, a decrease in the absorbance of the band related to free carbonyl and an increase in the hydrogen bonding carbonyl were observed. This finding indicates that free urethane groups transformed into the strong hydrogen-bonded urethane groups as a result of phase separation changes during the reprocessing [32]. Another visible change was the presence of a signal from carbon dioxide for both series of samples after the second processing (100H_2P and 90H_2P), which may indicate the presence of this compound in the measurement atmosphere.

### 3.2. Influence of Reprocessing of Bio-TPUs on Thermal and Thermomechanical Properties

#### 3.2.1. Thermogravimetric Analysis (TGA)

Polyurethanes belong to a group of materials, which are not highly thermally stable. The thermal stability of polyurethanes depends on the type of isocyanate and glycol used during the synthesis, as well as the symmetry, lengths, and concentrations of hard and soft segments [33,34]. Additionally, if we consider TPU processing, which is usually conducted at a high temperature and shear stress, these factors affect the thermal properties and should be taken into account. During the processing and reprocessing of the polymer chain scission, cross-linking (chemical and physical) or degradation (as a result of thermo-oxidation) might occur. As a result, noticeable changes occur in the average molecular weight, the molecular weight distribution, and the thermal/mechanical properties [27].

**Figure 5:**
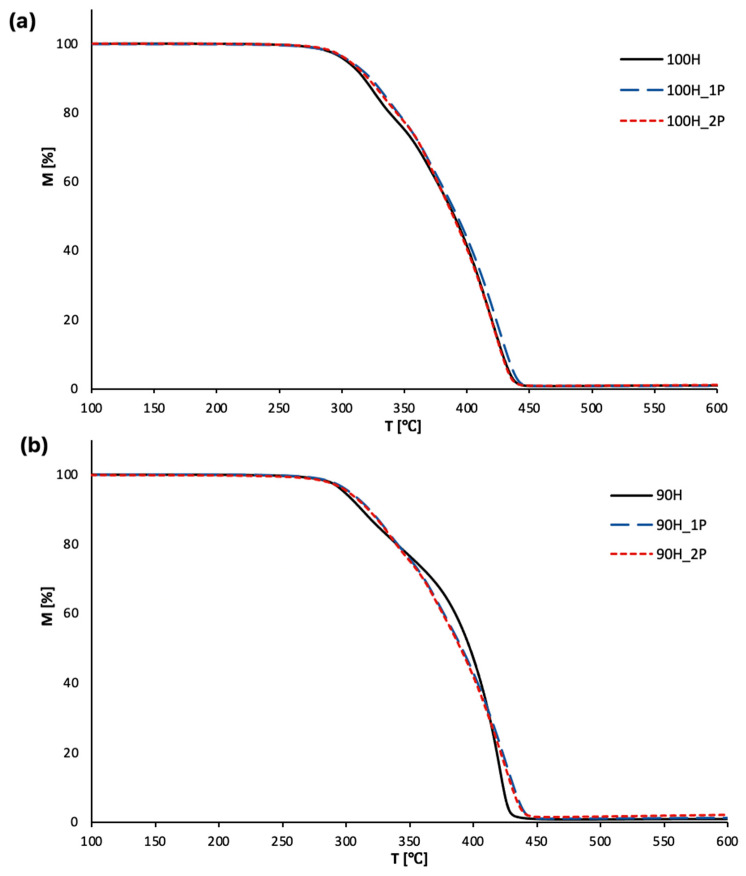
TG curves of bio-TPUs: (**a**) 100H series and (**b**) 90H series before and after two cycles of processing.

**Figure 6:**
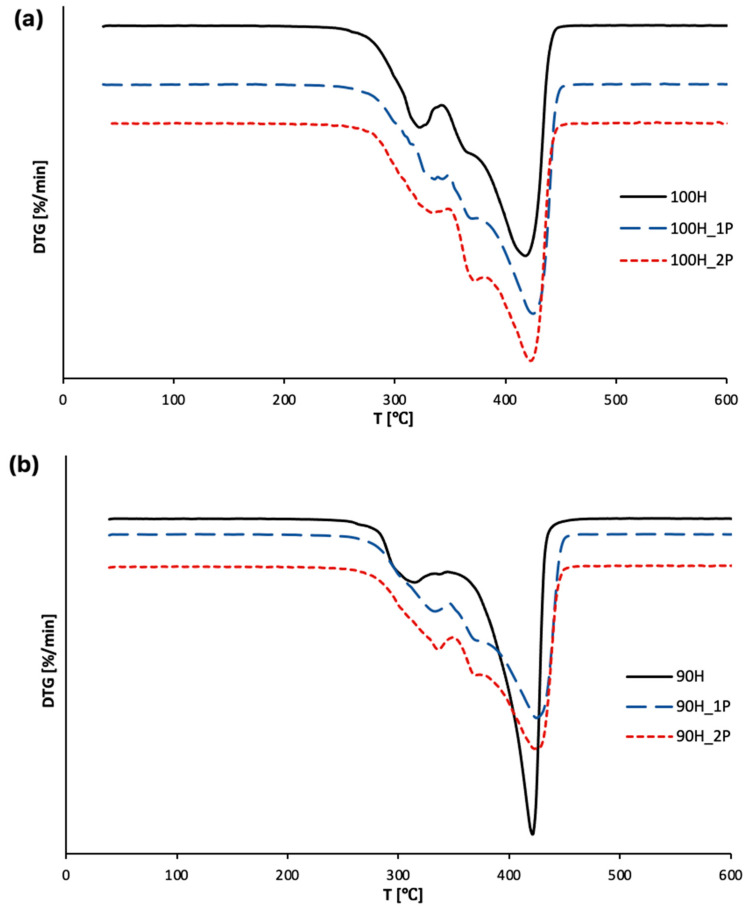
DTG curves of bio-TPUs: (**a**) 100H series and (**b**) 90H series before and after two cycles of processing.

A thermogravimetric analysis provides numerous information about the thermal stability of materials. In this work, the TGA was used to evaluate the thermal properties of the bio-TPUs before and after each reprocessing cycle. The results are presented as TG and DTG curves in Figure 5 and Figure 6, while Table 3 contains the main parameters determined during the measurements, e.g., temperature of 5%, 10%, and 50% of the mass loss of bio-TPUs before and after first and second reprocessing cycles. 

Figure 5a,b presents the mass loss as a function of temperature for the bio-TPUs based on HDI diisocyanate (100H series) and a mixture of HDI and FLO (90H series), respectively. As can be seen, the beginning of thermal degradation, with 5% of mass loss (T_5%_), occurred at approximately 303 °C for the non-processed 100H materials. The materials after reprocessing exhibited slightly higher values of the analyzed parameters (ca. 306 °C). This slight improvement in the thermal stability of the reprocessed bio-TPUs 100H series can be a result of the structural reorganization in the hard phase. The improvement in the thermal stability can be the effect of cross-linking between the polymer chains and primary degradation products, e.g., diisocyanates and diols.

Considering the properties of materials from the 90H series (Figure 5b), the thermal degradation of the non-processed material occurs at a lower temperature in comparison to the 100H series, and was noted at 298 °C. This phenomenon results from the chemical structure of the applied partially bio-based diisocyanate. The Tolonate X Flo 100 contains a side aliphatic chain, and on the other hand, is an allophanate type and partially isocyanate [35] with a lower thermal stability [36]. Single and double reprocessing cycles caused a slight increase in the T_5%_ to 303 and 302 °C, respectively. As in the case of the materials series 100H, a slight enhancement in the thermal stability might be a result of the structure reorganization and cross-linking that occurred in the hard phase. The residual mass of the polyurethanes at 600 °C was also determined from the TG curves. For both bio-TPU series, the highest residual mass at this temperature was observed for the materials that were reprocessed twice. If we consider processing and reprocessing, which were conducted at higher temperatures, with the presence of shear stress, it should be noted that bio-TPUs are subjected to themo-oxidative degradation during processing, and cross-linking may occur. As a result, a slightly higher ash residue at 600 °C was noted.

According to the literature, TPU degradation involves two main steps. The first step of degradation is related to the decomposition of hard segments, while the second step is the decomposition of soft segments [37,38]. By analyzing Figure 6a,b, the two-step degradation process was observed for both series of non-processed bio-TPU materials, whereas for materials after reprocessing, three-stage degradation was observed. 

The first step of thermal degradation is related to the degradation of hard segments. It is a result of the breaking of urethane bonds and compounds such isocyanates and polyols, and primary and secondary amines and carbon dioxide are formed [39,40]. The temperature of maximum degradation rate in the first step, T_max1_, was higher for the reprocessed TPUs, while DTG_max1_ gently decreased (see Table 3), which can be explained by structural changes during the material processing. Based on the obtained results, we notice that the thermal stability depends mainly on the hard segments’ compositions. In the second step of degradation, the breakage of the C-O-C bond promotes the degradation of the soft segments. The degradation of SS causes a decrease in the molecular weight and leads to an increase in the thermal degradation rate. If we consider the processed bio-TPUs, for both series, the soft segment degradation step is divided into two phases. It is probably related to the previously mentioned chain scission of bio-TPUs, which is built from soft segments. The temperature of the maximum degradation rate in the third step was approximately 421–425 °C.

#### 3.2.2. Differential Scanning Calorimetry

The thermal properties of polyurethanes are influenced by various factors, including the chemical structure of monomers, the symmetry and molecular weights of their chains, the molar ratios of the isocyanate and hydroxyl groups, the degree of phase separation, and the presence and type of hydrogen bonds [41]. On the one hand, urethane groups are able to form intramolecular, or particularly, intermolecular hydrogen bonds with ether groups derived from the structure of soft segments. Moreover, hydrogen bonds might be formed specifically from the urethane group with other urethane groups on other neighboring chains [42]. 

**Figure 7:**
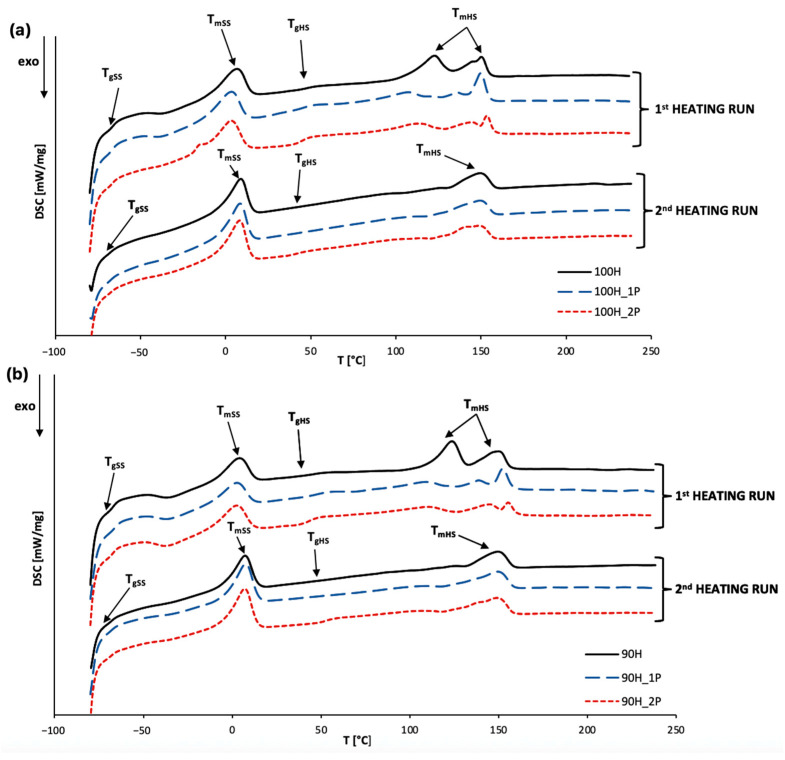
The endothermic curves of TPUs: a) 100H series and b) 90H series before and after two cycles of processing.

**Figure 8:**
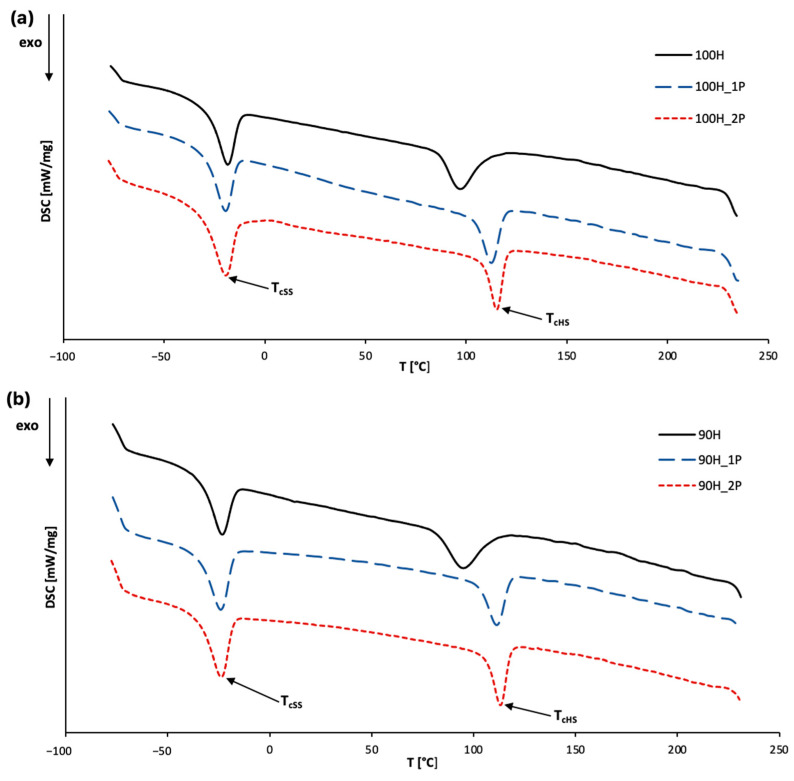
The exothermic curves of TPUs: (**a**) 100H series and (**b**) 90H series before and after two cycles of processing.

In this work, we focused on analyzing the impact of the chemical structure of hard segments (HS) and the number of reprocessing cycles on the thermal properties of bio-based thermoplastic polyurethanes (bio-TPUs). To achieve this, we performed differential scanning calorimetry (DSC) to evaluate the glass transition temperature (T_g_), melting behavior, and crystallization of the bio-TPUs. The endothermic and exothermic curves were obtained and are presented in Figure 7 and Figure 8, respectively, and all determined thermal parameters are listed in Table 4. 

The endothermic curves (Figure 7a,b) show characteristic thermal effects for the thermoplastic polyurethanes, indicating the glass transition temperature of the soft segments (T_gSS_) and hard segments (T_gHS_), as well as the melting of these two phases.

Based on the endothermic curves, we analyzed the influence of the number of reprocessing cycles and hard segments’ compositions on the material properties. Considering the first heating run, the presence of inhomogeneity in the size and content of ordered phases (both soft and hard) was observed, and it was especially noticeable in the case of hard segments. This could be attributed to the incorporation of partially bio-based isocyanates in the hard segments, leading to dispersion and miscibility of some hard segments in the soft phase. After the second heating run, single melting peaks for both soft and hard segments appeared, indicating ordering in soft segment microdomains. This can be explained by removing the thermal history of the sample in the first heating and structure reorganization. 

The melting point of soft segments (T_mSS_) determined based on the second heating run was in the ranges of 8.1–8.9 °C and 7.1–7.6 °C and was higher for the bio-TPU 100H series. For both series of materials, a slight decrease in the T_mSS_ and an increase in the melting enthalpy (ΔH_mSS_) of the soft segments after each reprocessing was visible. This finding is related with the ordering in the soft segments’ microdomains. It is worth mentioning that polyol used for synthesis is characterized by a linear structure and a tendency of crystallization and recrystallization.

For hard segments, the melting point (T_mHS_) determined based on the second heating run reached values between 149.1 and 149.9 °C for the bio-TPU 100H series. A slight decrease in T_mHS_ and ΔH_mHS_ with each subsequent reprocessing cycle could be observed. This might suggest a decrease in the crystallinity in the hard segments’ domains.

In the case of the bio-TPU 90H series, the HS melting point ranged between 149.7 and 150.0 °C (Table 3). Considering the melting enthalpy of the hard segments, a higher ΔH_mHS_ was registered with each subsequent reprocessing cycle, which indicated an increase in the crystallinity in the hard segments. This may be a result of chain scission during the reprocessing, and thereby, a higher mobility of a shorter chain is possible.

By analyzing the first heating run and the glass transition temperature of hard segments (T_gHS_), it was found that T_gHS_ had a range of 43.2–54.8 °C for the 100H series and was slightly lower for the materials in the 90H series. In the second heating run, T_gHS_ was observed for the twice-processed materials and was higher for the material 90H. The first heating and subsequent cooling led to the reorganization of the ordered phase, which was particularly noticeable in the case of hard segments, which explains the disappearance of T_gSS_ and T_gHS_ in the curves from the second heating run.

Figure 8 presents the exothermic curves, showing two exothermic peaks for each material. The first peak corresponds to the crystallization of soft segments, while the second one represents the crystallization of hard segments. The potential for crystallization and the formation of hard segment domains strongly depends on the HS structure, its type of hydrogen bonding, and chain symmetry [43].

The temperature in the crystallization points of SS were revealed to be in the range from −19.7 to −18.5 °C for the 100H series and from −23.5 to −24.1 °C for the 90H series, with insignificant changes in each subsequent reprocessing cycle. The enthalpy of crystallization (ΔH_cSS_) of soft segments mostly depends on the number of processing cycles, and increases after reprocessing, suggesting enhanced ordering in the soft phase. Regarding the crystallization of hard segments, the T_cHS_ revealed crystallization points between 96.8 and 115.1 °C (100H series) and 94.9–113.1 °C (90H series). Furthermore, the higher amount of bio-based diisocyanate in the HS structure resulted in higher T_cHS_ and lower ΔH_cHS_, indicating that reprocessing affects the crystallization behavior of bio-TPUs, leading to changes in the structure order and crystalline phase content.

#### 3.2.3. Thermomechanical Properties

**Figure 9:**
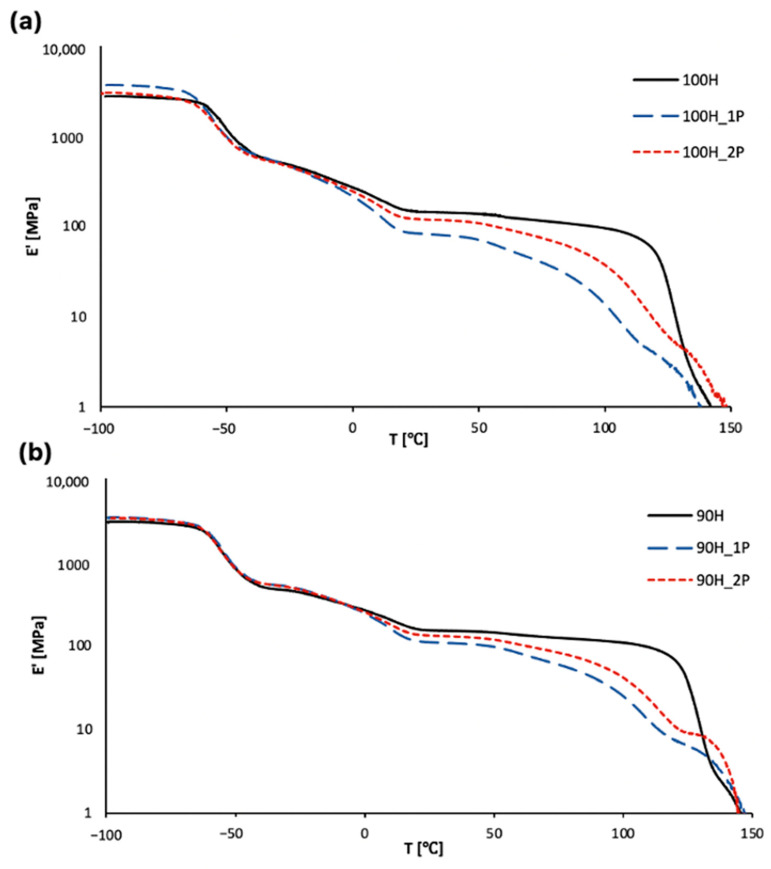
Temperature dependence of storage modulus for TPUs: (**a**) 100H series and (**b**) 90H series before and after two cycles of processing.

**Figure 10:**
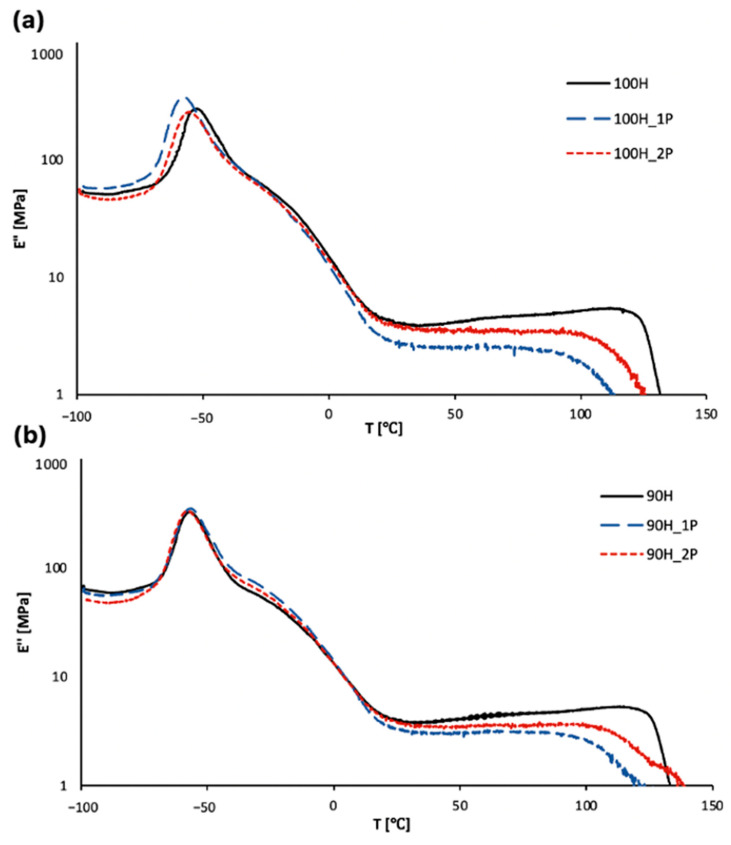
Temperature dependence of loss modulus for TPUs: (**a**) 100H series and (**b**) 90H series before and after two cycles of processing.

The temperature dependences of the storage modulus, loss modulus (E’’), and damping factor (tanδ) are presented in Figure 9 and Figure 10. The most important thermo-mechanical parameters using DMTA are summarized in Table 5. 

The storage modulus represents the amount of energy stored in the material, which can be recovered after deformation (elastic behavior), while the loss modulus is related to the amount of energy that is dissipated in the material during deformation (viscous behavior). The energy is dissipated due to molecular rearrangements [44]. The storage modulus is related to the stiffness of the material, and the highest values of this parameter are observed in a glassy state. The decrease in the storage modulus observed around −55 °C is related to the glass transition of soft segments and is connected with higher movement ability of polymer chains [45]. The second decrease in storage modulus at around 0 °C is related to the melting of soft segments, and the third one (very slight) above 60 °C is connected with the glass transition temperature of the hard segments, while the fourth one is related to the melting of the material. Similar behaviors were observed in other works [46,47]. At room temperature, where the materials are in a rubbery state, the values of the storage modulus are relatively low (in comparison to glassy state) and are in the range from 80 to 150 MPa. Similar trends were observed in both types of synthesized and processed bio-TPUs, i.e., increased values of the storage modulus in a glassy state in order (100H < 100H_2P < 100H_1P (and 90H < 90H_2P < 90H_1P)) and further decreased values in a rubbery state in order (100H > 100H_2P > 100H_1P (90H > 90H_2P > 90H_1P)). This shows that the significant microstructural change in the materials occurs in the materials during the first processing of the material (after the synthesis and final curing). The thermo-mechanical processing of the material results in the degradation of polymer chains (for example, the scission of main chains) and/or physical and chemical cross-links if they are presented in the materials, which was noticed in the TG results and tensile properties.

**Figure 11:**
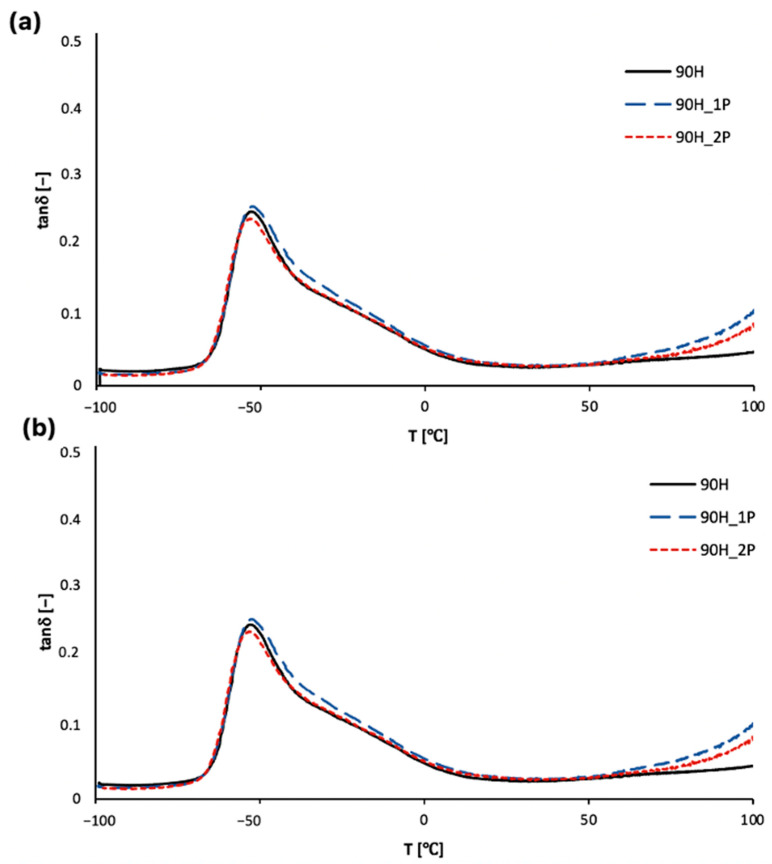
Temperature dependence of damping factor for TPUs: (**a**) 100H series and (**b**) 90H series before and after two cycles of processing.

The damping factor (tanδ) is defined as a ratio of the loss modulus to the storage modulus, and indicates the structural transitions in the material. The glass transition temperature is the temperature at which the damping factor reaches the maximum value, and in the case of thermoplastic bio-based polyurethanes, it is related to the glass transition of the soft segments (composed mainly of polyol PO3G moieties) [47,48,49]. The transformation from the glassy state into the rubbery state occurs in a similar temperature range from −49 to −53 °C, but lower values were generally observed for the bio-TPUs synthesized using a diisocyanate mixture (90H), which is visible in Figure 11. The addition of partially bio-based diisocyanates to the bio-TPU 90H series resulted in a slight decrease in the glass transition temperature as a result of a higher mobility of polymer chains, which was probably due to the presence of a side chain in the chemical structure of the mentioned diisocyanate. The tanδ is related to the ability of the material to dissipate the energy of deformation and damping properties. The better performance in this matter was observed for 90H bio-TPUs, but the differences between both types of materials were very slight. The number of processing cycles also did not significantly affect the damping properties of the materials.

### 3.3. Influence of Reprocessing of Bio-TPUs on the Processing Properties

#### Melt Flow Index (MFI)

The processing properties of both the reference and reprocessed bio-TPUs were investigated by the determination of the mass flow rate (MFR) and melt volume flow rate (MVR). The melt flow index (MFI) is a crucial factor influencing the melt processability and mainly depends on the polymer structure, number, average molecular weight, and polydispersity index [47]. In this work, the MFR and MVR were determined to control the changes that may occur in the material during the reprocessing cycles. The processing properties determined for the bio-TPUs (100H and 90H series) are summarized in Table 6. 

Depending on the chemical composition of the bio-TPUs’ hard segments, either a decrease or an increase in the MFR was observed. The materials obtained using a single diisocyanate exhibited a decrease in the MFR with an increasing number of reprocessing cycles, while the opposite phenomenon was observed for the bio-TPUs based on the diisocyanate mixtures. The decreased MFR and MFI values for the bio-TPU 100H series might be a consequence of the partial degradation of the bio-TPU chain and some structural reorganization (branching or cross-linking structure) due to each subsequent reprocessing cycle [32]. In the case of the bio-TPU 90H series, increased MFI values suggest the degradation of the polymer chain, and thereby the molecular weight of the macromolecules. When evaluating the melt density (ρ_m_), we observed a rising trend with an increase in the number of processing cycles, regardless of the bio-TPUs’ chemical composition. This increase in ρ_m_ suggests morphological changes, physical or chemical cross-linking, chain scission, and general structural reorganization of the bio-TPU. By comparing the melt density of the materials in the 100H and 90H series, we observed a higher value for the bio-TPU based on the diisocyanate mixture, indicating that the materials in the 90H series exhibited a higher content of the amorphous phase.

### 3.4. Correlation between Number of Reprocessing Cycles and Mechanical and Physical Characteristic of Bio-TPUs

#### Tensile Properties, Hardness, and Density

**Figure 12:**
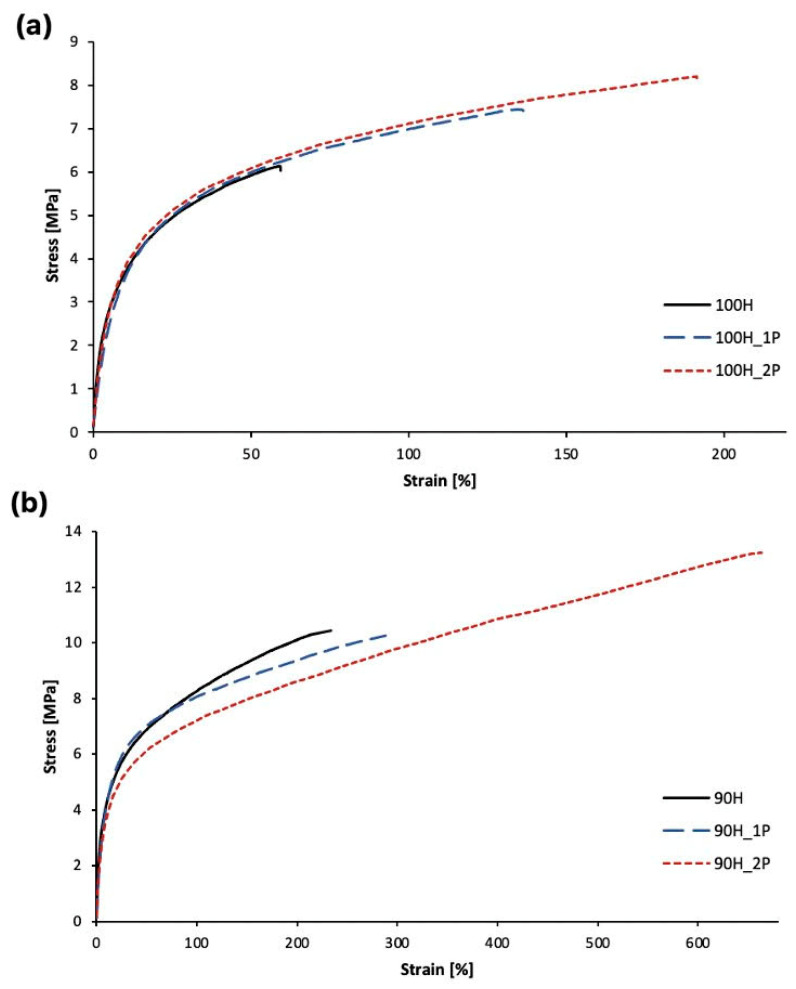
The stress–strain curves for TPUs: (**a**) 100H series and (**b**) 90H series before and after two cycles of processing.

As is known, the processing of polymeric materials might cause irreversible thermal and mechanical degradation, leading to changes in the properties of recycled material [13]. Static tensile tests, hardness, and density measurements were carried out to determine the relationship between the number of reprocessing cycles and the mechanical and physical properties of bio-TPUs. The values of the determined parameters such as the tensile strength (TS_b_), elongation at break (ε_break_) and permanent elongation (ε)), hardness (H), and density (ρ) are shown in Table 7, while the exemplary stress–strain curves are presented in Figure 12.

By analyzing Table 7, we can observe the differences in the tensile properties between the non-processed series of the bio-TPUs. The materials based on a mixture of isocyanates (90H series) have higher values of TSb (increase of about 4 MPa) compared to the materials in the 100H series. Additionally, the elongation at break (εb) and the permanent elongation (εt) of the 90H material are significantly higher by approximately 178% and 43.1%, respectively, compared to the corresponding values of the 100H material. These differences may be attributed to the nature and branching in the structure of the partially bio-based diisocyanate, which imparts mobility to the hard segments and increases the content of hard segments (calculated as wt.%).

The tensile properties increase after the first and second processing cycles in both the 100H and 90H series. The differences are noticeable and demonstrate that the material achieves a higher tensile strength and elongation at break after each reprocessing cycle. For example, in the case of the TPU 100HDI series, the material after a single processing cycle exhibits higher tensile strength by approximately 1.4 MPa and higher elongation at break by about 88% compared to the non-processed material. These changes can be explained by the structural reorganization that occur in both the soft and hard segments. It can be caused by a change in the degree of phase separation, which results from the chain scission in the hard segments (HS). Considering the 90H series, a similar trend is observed. This finding might be supported by the DSC results. Morphological changes in the bio-TPUs structures, as the result of chain scission or branching, lead to an increase in the chain mobility, which is visible as changes in the εb results. 

The results of hardness testing also suggest changes in the bio-TPUs. The highest hardness values are visible for the non-processed materials, and the hardness decreases with an increase in the number of processing cycles. Again, multiple processing might result in the morphological changes and weaken the intermolecular forces. For the materials coded as 100H, the hardness ranges from 88.6 to 91.7 °ShA, and for the materials in the 90H series, it ranges from 87.6 to 92.8 °ShA. The slightly higher value for the reference materials in the 90H series may be due to the higher content of HS in this material. In general, the hardness values of the 100H and 90H series are at a similar level and differ marginally. Similar findings were described by Głowińska, Cybart, and Datta [20]. The densities of all materials are quite similar and higher than 1.0 g/cm³, ranging from 1.059 to 1.120 g/cm³. These are typical values for thermoplastic polyurethanes. Slight differences in density can be observed in the case of materials after a single processing cycle, suggesting changes in the structure or morphology of bio-TPUs. 

## 4. Conclusions

In this study, we analyzed the influence of both the hard segment structure and number of reprocessing cycles of bio-TPUs on their thermal, thermomechanical, mechanical, and processing properties. Our results demonstrate that the changes observed in most cases were insignificant, indicating the advantage of easily recyclable polymeric materials. The multiple processing of bio-TPUs did not lead to a significant lowering in the important properties, making them suitable candidates for mechanical recycling. 

The FTIR analysis provided insights into the chemical structures of bio-TPUs, indicating changes in the range of carbonyl vibration and the hydrogen bonding content, particularly in the hard segments. The thermal stability of the bio-TPUs was found to be primarily dependent on the composition of the hard segments and the number of reprocessing cycles. The addition of partially bio-based isocyanates to the structure of hard segments resulted in a slight decrease in the temperature of 5% mass loss (T_5%_) of the bio-TPUs. The DSC measurements allowed us to study the thermal behavior and segmented structure of the bio-TPUs and allowed us to detect the morphological reorganization as a result of each reprocessing cycle. Interestingly, the melt flow properties (MFR and MFI) showed contrasting trends between the 100H series and the 90H series, with the MFR and MFI values decreasing with each reprocessing cycle for the 100H series, and increasing for the 90H series. A noticeable effect of reprocessing was revealed in the mechanical properties. The tensile strength increased with each subsequent reprocessing cycle from 6.1 to 8.2 for the 100H series and from 10.1 to 13.2 MPa for the 90H series. Also, the elongation at break significantly increased from 57 to 235%, and from 191 to 663% for the 100H and 90H series, respectively. Multiple processing resulted in a decrease in the hardness of the bio-TPU (by about 3–4 °ShA) for both material series, which was a consequence of the structure reorganization.

In summary, our work sheds light on the potential of using bio-TPUs for sustainable development and waste reduction. The ability to reprocess these materials without a significant loss of properties opens avenues for reducing the environmental impact and promoting circular economy practices. However, it is essential to acknowledge the limitations of this study, such as the need for further investigation into the long-term stability of reprocessed bio-TPUs.

## Figures and Tables

**Table 1:** Codes of the samples (including the weight contents of isocyanate groups introduced by HDI in the prepolymerization reaction), their description, and contents of hard segments and bio-based components.

Sample	Description	HS Content (wt.%)	Bio Components Content (wt.%)	Pictures of Bio-TPUs
100H	100 wt.% of NCO groups derived from HDI Raw bio-TPU (non-processed)	28.3	78.4	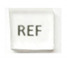
100H_1P	100 wt.% of NCO groups derived from HDI Bio-TPU after 1st cycle of processing			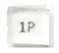
100H_2P	100 wt.% of NCO groups derived from HDI Bio-TPU after 2nd cycle of processing			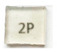
90H	90 wt.% of NCO groups derived from HDI 10 wt.% of NCO from Tolonate X FLO 100 Raw bio-TPU (non-processed)	30.3	78.8	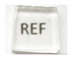
90H_1P	90 wt.% of NCO groups derived from HDI 10 wt.% of NCO from Tolonate X FLO 100 Bio-TPU after 1st cycle of processing			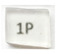
90H_2P	90 wt.% of NCO groups derived from HDI 10 wt.% of NCO from Tolonate X FLO 100 Bio-TPU after 2nd cycle of processing			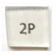

**Table 2:** The assignment of absorption bands to chemical groups for the synthesized bio-TPUs after 100H and 90H.

Bond	Samples						
	Reference [23]	100H	100H_1P	100H_2P	90H	90H_1P	90H_2P
	Wavenumber, (cm^−1^)						
N-H	3430	3315	3320	3313	3313	3318	3310
C-H_asym._	2900–2960	2915	2930	2927	2919	2923	2920
C-H_sym._	2870–2890	2850	2860	2852	2848	2852	2850
O=C=O	2350	-	-	2343	-	-	2350–2260
C=O	1700–1770	1683, 1725	1683, 1727	1683, 1712	1683, 1714	1683, 1724	1683, 1720
C-N	1530–1580	1533	1540	1529	1531	1531	1530
C-O-C	1080–1135	1097	1100	1106	1095	1099	1100
C-H_def_	635–1000	630–779	634–777	632–779	628–790	622–777	625–762

**Table 3:** Thermal properties such as temperature of 5, 10 and 50% mass loss, temperature of non-processed bio-TPUs and after two reprocessing cycles.

Sample	Temperature of 5, 10 and 50% Weight Loss (°C)			Temperature at DTG Maximum (°C)			DTG (%/min)			Mass at 600 °C (%)
	T5%	T10%	T50%	T_max1_	T_max2_	T_max3_	DTG_1_	DTG_2_	DTG_3_	
100H	304	318	390	325	-	421	−5.2	-	−11.7	0.94
100H_1P	306	323	392	336	371	425	−4.8	−6.9	−11.6	0.83
100H_2P	306	321	389	334	372	423	−4.5	−8.0	−12.1	1.09
90H	298	312	397	315	-	421	−4.0	-	−19.7	0.91
90H_1P	303	318	391	333	368	424	−4.8	−6.6	−11.5	1.25
90H_2P	302	318	390	336	369	423	−5.2	−6.8	−11.4	2.17

**Table 4:** Thermal properties of bio-TPUs determined using DSC technique.

	Sample											
	100H		100H_1P		100H_2P		90H		90H_1P		90H_2P	
	I Run	II Run	I Run	II Run	I Run	II Run	I Run	II Run	I Run	II Run	I Run	II Run
T_gSS_ (°C)	−65.7	−60.5	−66.4	-	−66.7	−67.7	−70.4	−61.3	−66.2	-	−68.7	−63.8
T_mSS_ (°C)	6.2	8.9	2.8	8.3	2.5	8.1	3.8	7.1	2.1	7.6	7.1	7.1
ΔH_mSS_ (J/g)	17.9	19.5	20.6	21.7	22.7	21.7	16.0	19.0	16.2	22.9	18.9	22.3
T_gHS_ (°C)	48.0	-	54.8	-	43.2	37.0	52.7	-	52.7	-	43.2	52.7
T_mHS1_ (°C)	122.2	-	107.0	-	110.9	-	123.7	-	109.6	-	109.9	-
ΔH_mHS1_ (J/g)	11.4	-	2.6	-	5.2	-	13.4	-	2.8	-	4.0	-
T_mHS2_ (°C)	150.7	149.6	149.9	149.5	153.8	149.1	150.4	150.0	138.4	149.7	155.5	149.7
ΔH_mHS2_ (J/g)	7.1	12.8	10.0	11.4	7.5	10.7	9.2	9.9	4.5	13.2	8.3	13.2
T_cSS_ (°C)	−18.5	-	−19.3	-	−19.7	-	−23.5	-	−24.1	-	−24.0	-
ΔH_cSS_ (J/g)	−17.6	-	−17.8	-	−22.5	-	−15.3	-	−20.5	-	−18.9	-
T_cHS_ (°C)	96.8	-	112.4	-	115.1	-	94.9	-	111.2	-	113.1	-
ΔH_cHS_ (J/g)	−16.4	-	−14.5	-	−12.2	-	−17.8	-	−14.0	-	−14.4	-

**Table 5:** Thermomechanical properties of synthesized and processed bio-TPUs.

Sample	E′ at −100 °C (MPa)	E′ at 25 °C (MPa)	E″ (MPa)	tanδ (-)	T_gSS_ (°C)
100H	2790	148.7	273	0.207	−48.9
100H_1P	3650	82.9	346	0.217	−52.5
100H_2P	3010	120.6	256	0.203	−50.9
90H	3027	150.6	329	0.246	−52.9
90H_1P	3409	108.9	350	0.254	−52.7
90H_2P	3344	131.2	334	0.236	−53.3

**Table 6:** Processing parameters as MFR and MVR values and melt density (ρ_m_) of bio-TPUs.

Sample	T (°C)	Load (kg)	MFR (g/10 min)	MVR (cm^3^/10 min)	* δ_m_ (g/cm^3^)
100H	144	2.16	23.3 ± 3.9	29.7 ± 7.8	0.785
100H_1P	144	2.16	9.3 ± 0.3	9.4 ± 0.3	0.989
100H_2P	144	2.16	2.9 ± 0.7	2.9 ± 0.6	1.000
90H	155	5	15.6 ± 0.1	15.9 ± 0.1	0.977
90H_1P	150	2.16	43.2 ± 2.9	43.8 ± 2.1	0.986
90H_2P	150	2.16	39.1 ± 4.1	37.9 ± 2.3	1.032

Melt density (δ_m_) * at measurement temperature was calculated based on the following equation: ρm = MFR/MVR.

**Table 7:** Tensile properties, hardness, and density of bio-TPUs.

Sample	TS_b_ (MPa)	ɛ_b_ (%)	ɛ_t_ (%)	H °ShA	ρ (g/cm^3^)
100H	6.1 ± 0.2	57 ± 5	7.1 ± 0.4	91.7 ± 0.6	1.075 ± 0.002
100H_1P	7.4 ± 0.5	136 ± 15	25.3 ± 8.5	89.2 ± 0.3	1.059 ± 0.001
100H_2P	8.2 ± 0.3	191 ± 10	43.2 ± 13.2	88.6 ± 0.7	1.085 ± 0.014
90H	10.1 ± 0.3	235 ± 15	50.2 ± 15.9	92.8 ± 0.3	1.083 ± 0.001
90H_1P	10.9 ± 9.4	271 ± 13	71.5 ± 7.8	87.6 ± 0.5	1.120 ± 0.002
90H_2P	13.2 ± 0.4	663 ± 24	178.7 ± 5.9	88.7 ± 0.7	1.086 ± 0.011

## Data Availability

Not applicable.
